# Evolution of Antimicrobial Consumption During the First Wave of COVID-19 Pandemic

**DOI:** 10.3390/antibiotics10020132

**Published:** 2021-01-29

**Authors:** Santiago Grau, Daniel Echeverria-Esnal, Silvia Gómez-Zorrilla, Maria Eugenia Navarrete-Rouco, Joan Ramon Masclans, Merce Espona, Maria Pilar Gracia-Arnillas, Xavier Duran, Merce Comas, Juan Pablo Horcajada, Olivia Ferrández

**Affiliations:** 1 Pharmacy Department, Infectious Pathology and Antimicrobials Research Group (IPAR), Institut Hospital del Mar d’Investigacions Mèdiques (IMIM), Hospital del Mar, Parc de Salut Mar, Passeig Maritim 25-29, 08003 Barcelona, Spain; dechevarria@psmar.cat (D.E.-E.); mnavarrete@psmar.cat (M.E.N.-R.); mespona@psmar.cat (M.E.); oferrandez@psmar.cat (O.F.); 2 Medicine Department, Campus UAB, Bellaterra, Universitat Autònoma de Barcelona, Plaça Cívica, 08193 Barcelona, Spain; jrmasclans@psmar.cat; 3 Infectious Diseases Department, Hospital del Mar, Infectious Pathology and Antimicrobials Research Group (IPAR), Institut Hospital del Mar d’Investigacions Mèdiques (IMIM), Universitat Autònoma de Barcelona (UAB), CEXS—Universitat Pompeu Fabra, Passeig Maritim 25-29, 08003 Barcelona, Spain; sgomezzorrilla@psmar.cat (S.G.-Z.); jhorcajada@psmar.cat (J.P.H.); 4 Critical Care Department, GREPAC, IMIM (Mar Hospital Medical Research Institute), Hospital del Mar, Passeig Maritim 25-29, 08003 Barcelona, Spain; mgraciaa@psmar.cat; 5 Scientific, Statistics and Technical Department, Hospital del Mar-IMIM, Parc de Salut Mar, Passeig Maritim 25-29, 08003 Barcelona, Spain; xduran@imim.es; 6 Epidemiology and Evaluation, Research Network on Health Services in Chronic Diseases (REDISSEC), Hospital del Mar, Parc de Salut Mar, Passeig Maritim 25-29, 08003 Barcelona, Spain; mcomas@psmar.cat

**Keywords:** antimicrobial stewardship, COVID-19, defined daily doses (DDD), antimicrobial resistance, antibiotic consumption

## Abstract

*Background:* The first wave of COVID-19 pandemic may have significantly impacted antimicrobial consumption in hospitals. The objective of this study was to assess the evolution of antimicrobial consumption during this period. *Methods*: A retrospective quasi-experimental before–after study was conducted in a Spanish tertiary care hospital. The study compared two periods: pre-pandemic, from January 2018 to February 2020, and during the COVID-19 pandemic from March to June 2020. Antimicrobial consumption was analyzed monthly as defined daily doses (DDD)/100 bed-days and overall hospital and ICU consumption were evaluated. *Results:* An increase in the hospital consumption was noticed. Although only ceftaroline achieved statistical significance (*p* = 0.014), a rise was observed in most of the studied antimicrobials. A clear temporal pattern was detected. While an increase in ceftriaxone and azithromycin was observed during March, an increment in the consumption of daptomycin, carbapenems, linezolid, ceftaroline, novel cephalosporin/β-lactamase inhibitors or triazoles during April–May was noticed. In the ICU, these findings were more evident, namely ceftriaxone (*p* = 0.029), carbapenems (*p* = 0.002), daptomycin (*p* = 0.002), azithromycin (*p* = 0.030), and linezolid (*p* = 0.011) but followed a similar temporal pattern. *Conclusion*: An increase in the antimicrobial consumption during the first wave of COVID-19 pandemic was noticed, especially in the ICU. Availability of updated protocols and antimicrobial stewardship programs are essential to optimize these outcomes.

## 1. Introduction

Due to the concerns about the increase in antimicrobial resistance, a series of initiatives have been proposed to try to alleviate this problem. Hence, the development of antimicrobial stewardship programs (ASPs) is one of the most important objectives of various governments and the World Health Organization (WHO) [1,2,3,4]. One of the strategies of the ASPs is to carry out an exhaustive monitoring of antimicrobial consumption [5]. For this purpose, among the different measures, defined daily doses (DDD)/100 bed-days stand out as the most common source of information [5].

The appearance of an outbreak caused by a specific microorganism may be the cause of a marked increase in antimicrobial consumption. In the case of the Coronavirus Disease-19 (COVID-19) pandemic, an important impact on the DDD/100 bed-days of specific antibiotics could be expected [6]. Potential causes include an increase in hospital stay associated with this infection or the high rate of intensive care unit (ICU) admission, which in fact led to the collapse of beds for the care of critical patients. However, one of the main reasons would be the potential overprescription of antimicrobials due to concerns about bacterial co-infection. This trend is derived from influenza, an infection in which a high prevalence (58%) of co-infection has been demonstrated [7], mainly due to *Staphylococcus aureus*, *Streptococcus pneumoniae*, and *Streptococcus pyogenes* [8]. The available evidence nevertheless questions this practice and has shown a lower incidence of these co-infections compared to influenza [7,9]. In fact, there were not only differences in incidence, but also in the bacterial co-pathogen profiles, as in patients diagnosed with COVID-19, the commonest bacteria were *Mycoplasma pneumoniae*, *Pseudomonas aeruginosa*, and *Haemophilus influenzae* [9].

These findings would suppose that most of the prescribed antibiotics for COVID-19 would be therefore unnecessary. This overuse carries potential negative effects, namely a higher risk of antimicrobial resistance [10]. Some experts have already expressed their concerns over the potential influence that this pandemic may have on accelerating the threat of antimicrobial resistance [11]. Based on these data, the WHO and other experts recommend that antibiotic therapy should not be initiated for suspected or confirmed mild COVID-19, whereas for moderate COVID-19, antibiotics should not be prescribed unless a high clinical suspicion of bacterial infection is present or in critically-ill patients [12,13,14].

Other studies have shown an increase in the consumption of antimicrobials during the first wave of the COVID-19 pandemic [6,15,16,17]. However, these studies either have not described the data according to the different molecules or have not performed a statistical analysis of the data.

The objective of this study was to analyze the evolution of DDD/100 bed-days per month over a two-year pre-pandemic period and compare it with the first wave of the COVID-19 pandemic.

## 2. Results

During the pre-pandemic phase, the hospital registered a total of 49,069 admissions with a monthly rate of 1887 admissions. During the pandemic, the crest of the wave happened on 1 April 2020, when 386 patients diagnosed with COVID-19 were hospitalized. The average monthly admission rate during this period was 2143 admissions.

**Figure 1:**
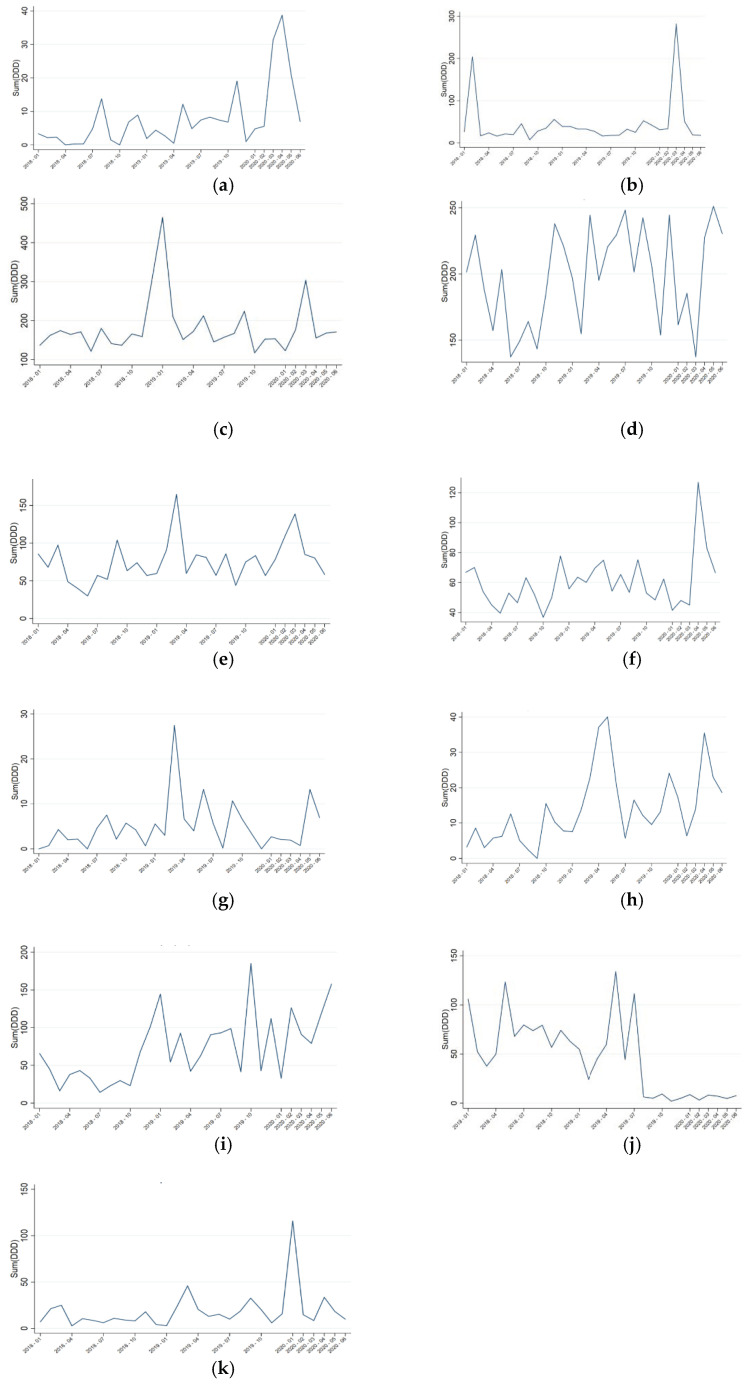
Evolution of drug consumption in hospital expressed in defined daily doses (DDD)/100 bed-days. (**a**) Ceftaroline: β-change 11.771, *p* = 0.014; (**b**) azithromycin: β-change 63.129, *p* = 0.085; (**c**) ceftriaxone: β-change 18.390, *p* = 0.692; (**d**) carbapenems: β-change −12.714, *p* = 0.592; (**e**) triazoles: β-change 17.601, *p* = 0.333; (**f**) linezolid β-change 13.725, *p* = 0.211; (**g**) liposomal amphotericin B: β-change −2.692, *p* = 0.463; (**h**) novel cephalosporins/β-lactamase inhibitors: β-change −4.064, *p* = 0.491; (**i**) daptomycin: β-change 11.147, *p*= 0.644; (**j**) vancomycin: β-change −7.435, *p* = 0.705; (**k**) echinocandins: β-change −20.550, *p* = 0.117.

The overall hospital antimicrobial consumption by molecules is described in Figure 1. A statistically significant increase was only observed for ceftaroline. Despite not reaching statistical significance, an important rise in the consumption of ceftriaxone, azithromycin, carbapenems, daptomycin, novel cephalosporins/β-lactamase inhibitors, linezolid, triazoles, and liposomal amphotericin B was noticed.

A striking temporary pattern was observed. Whereas ceftriaxone, azithromycin, and triazoles reached their maximum consumption in March, the rest of the antimicrobials, especially those with a broader spectrum, achieved their peaks in April–May.

**Figure 2:**
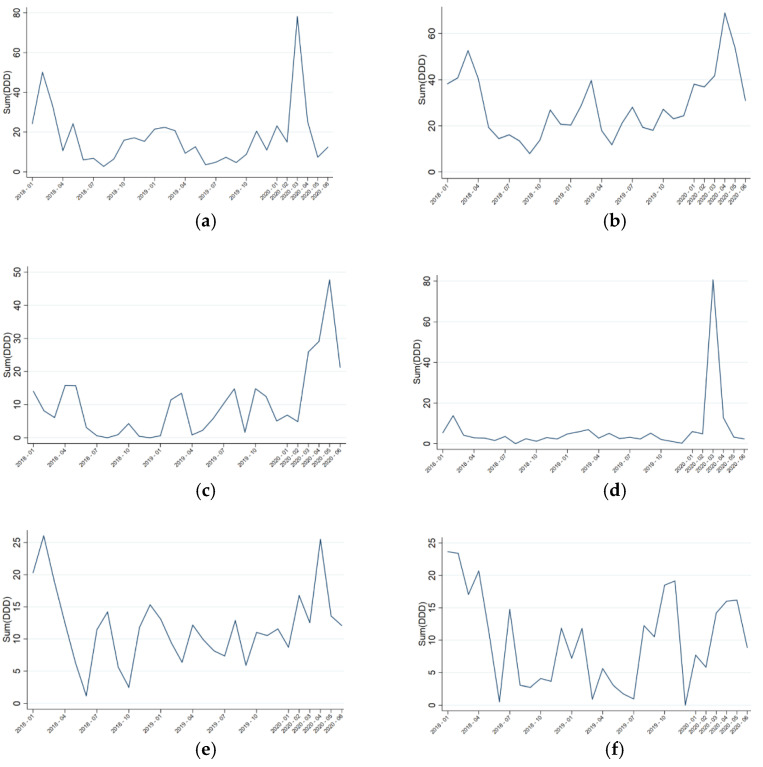

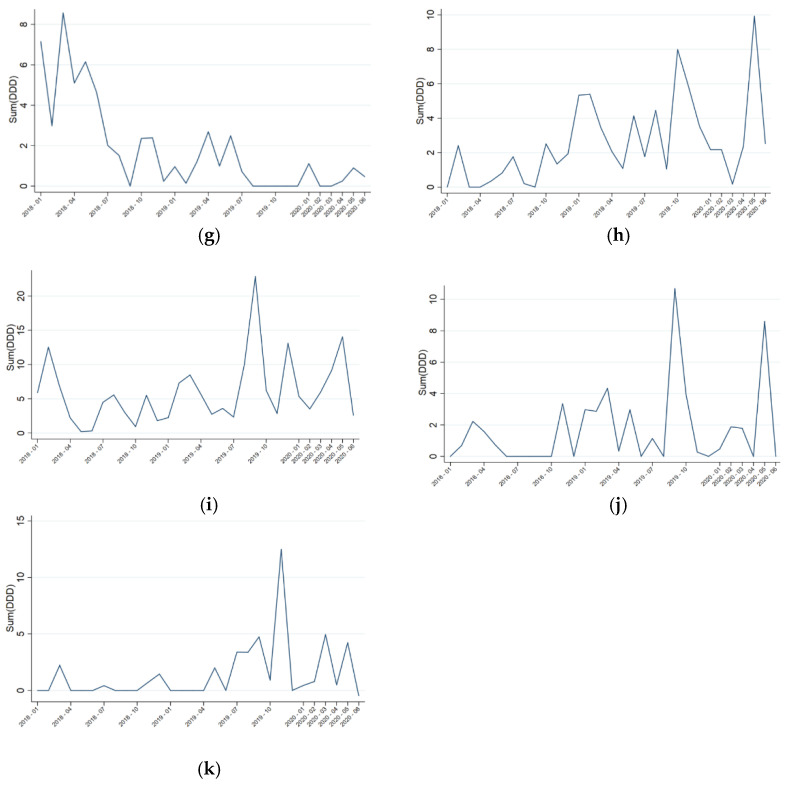
Evolution of drug consumption in the expanded-intensive care unit (ICU) expressed in defined daily doses (DDD)/100 bed-days. (**a**) Ceftriaxone: β-change 21.501, *p* = 0.029; (**b**) carbapenems: β-change 26.103, *p* = 0.002; (**c**) daptomycin: β-change 18.093, *p* = 0.002; (**d**) azithromycin: β-change 19.422, *p* = 0.030; (**e**) linezolid: β-change 9.183, *p* = 0.011; (**f**) triazoles: β-change 7.939, *p* = 0.089; (**g**) vancomycin: β-change 1.816, *p* = 0.079; (**h**) novel cephalosporins/β-lactamase inhibitors: β-change −1.698, *p* = 0.225; (**i**) echinocandins: β-change −1.525, *p* = 0.626; (**j**) liposomal amphotericin B: β-change −0.311, *p* = 0.852; (**k**) ceftaroline: β-change −1.550, *p* = 0.339.

When the DDD/100 bed-days were analyzed in the expanded-ICU, significant differences were observed for ceftriaxone, carbapenems, daptomycin, azithromycin, and linezolid, with a non-statistically significant upward trend for triazoles and vancomycin (Figure 2). Similar to what was observed in the overall hospital consumption, ceftriaxone and azithromycin reached their maximum consumption in March, while the extended-spectrum molecules reached it in April–May. Compared to the previous analysis, in the ICU a higher increase in daptomycin and vancomycin was observed, probably because of a higher incidence of catheter-related bloodstream infections.

None of the antimicrobials studied showed a decrease in their use when the pre-pandemic and pandemic periods were compared.

**Figure 3:**
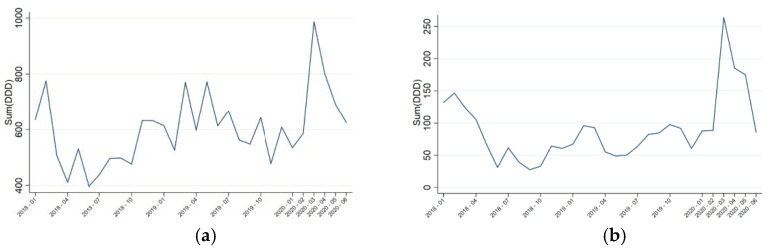
Evolution of global antimicrobial consumption expressed in defined daily doses (DDD)/100 bed-days. (**a**) Hospital: β-change 124.242, *p* = 0.106; (**b**) extended-intensive care unit (ICU): β-change 97.960, *p* = 0.001.

The evolution of global antimicrobial consumption both in the hospital and in the ICU was described in Figure 3. In both areas, an increase in antimicrobial consumption was noticed, although a statistically significant different could only be observed in the ICU. Strikingly, in March the antimicrobial use record was broken. Even if antibiotic prescription decreased in the upcoming months, a worrisome consumption was still detected, especially in the ICU.

## 3. Discussion

The present study shows an increase in the global hospital prescription of antimicrobials during the first wave of COVID-19 pandemic, with a clear temporary pattern.

During the first part of the pandemic period in March, a rise in the consumption of molecules included in the treatment protocols for the treatment of COVID-19, such as ceftriaxone and azithromycin, was noticed. In some of them, although a marked rise was observed, no statistical difference was obtained, probably due to the high consumption in the previous period.

Of interest, in subsequent months (April–May), an increase in the consumption of extended-spectrum drugs was observed, which may be related to an increment in the device-related (mainly catheter-related bloodstream infections) and superinfections.

Our data are consistent with those from a Spanish study where an initial increase in DDD/100 bed-days of amoxicillin–clavulanate was observed, a drug that was used in a similar way to the ceftriaxone in our study [16]. According to data on the prevalence of co-infections [18,19,20], including in Spain [21,22], most of this consumption is unnecessary.

A recent rapid review and meta-analysis including 154 studies with available data from 30,623 patients showed that the prevalence of antibiotic prescribing was 74.6%, whereas estimated bacterial co-infection was 8.6% [23]. Other systematic reviews and meta-analyses showed similar results, and established that 71.9% of patients admitted with COVID-19 received antibiotics, although bacterial co-infection prevalence at presentation was 3.5% (95% confidence interval (CI), 0.4–6.7%) [19,21]. According to a survey completed by 166 participants from 23 countries, clinical presentation was the most important reason for initiating antibiotic therapy [24]. Most of them rated as the highest need the coverage of atypical microorganisms, followed by *S. aureus* [24]. The consequences of this excessive use are well-known, with a higher risk of adverse events or selection of resistant bugs. In fact, among other factors, this excessive empiric use may have contributed to the increase in the consumption of extended spectrum antibiotics used for the treatment of superinfections.

These findings were more evident in the expanded-ICU but followed a similar pattern. The increase in DDD/100 bed-days observed in drugs such as carbapenems or echinocandins could be related to the treatment of superinfections. In fact, the estimated prevalence of superinfections in these patients during the pandemic were 14% (95% CI, 5–26%) [18], which is greater than that observed in hospital wards [20]. The same study described a rate of bacteremia in the ICU of 25% after 15 days of admission, a situation that would justify the increase in DDD/100 bed-days observed in our study for daptomycin or vancomycin parallel to the increase in bacteremia that occurred in our ICU during the pandemic phase (data not shown) [18]. The uses of immunosuppressive drugs such as corticosteroids or tocilizumab and the fact that non-specialized health personnel were working in these wards may play a role in these findings.

The use of fluconazole and echinocandins also increased during the studied period, which may have been due to their empiric use for candidemia. However, one study showed that no variation was observed in its incidence between the period before the pandemic and the first wave of COVID-19 [6]. An increase in the prescription of mold active molecules was also noticed, which may be associated with a rise in the diagnosis of invasive aspergillosis in severe patients with acute respiratory distress syndrome and COVID-19 [25].

There are other reasons that also may explain the increase in the use of antimicrobials during the COVID-19 pandemic, namely the initial ignorance of how to deal with this infection; the overflow of hospitals in terms of number of patients; the shortage of doctors with skills to deal with this situation; the lack of initial therapeutic protocols; the decrease in the activity of the ASP team; and, finally, the suspicion of bacterial co-infections or superinfections in patients with a prolonged hospital stay.

All this evidence underscores the vital role of ASPs in optimizing the use of antimicrobials within hospitals, even more in these special situations such as the COVID-19 pandemic. An interesting perspective highlighted the need for ongoing ASPs during COVID-19 and provided valuable recommendations [26]. An appropriate use of microbiological tests before the initiation of empiric antibiotic therapy, the promotion of local guidelines, the early de-escalation or discontinuation of therapy when clinical markers are not suggestive of bacterial co-infection, the guidance of antibiotic choice based on microbiological tests results, the early switch from intravenous to oral route, the limitation of the duration of antibiotic treatment to five days, and careful monitoring for potential drug interactions or toxicity are essential to improve antibiotic use during the next waves of the COVID-19 pandemic [26]. The use of biomarkers such as procalcitonin or C-reactive protein may also be a tool to optimize antibiotic prescribing in this setting, although available evidence is yet of low quality [27]. A recently published study demonstrated that a baseline white cell count > 8.2 × 10^6^ cells/mL or falling C-reactive protein could exclude bacterial co-infection in up to 46% of COVID-19 patients, which could facilitate antimicrobial stewardship efforts [28].

The present study is not exempt from limitations. A comparison of the incidences of the different infections in the compared periods would have been of interest for a more precise justification of the remarked observations or of the possible increase in the resistance rate of specific microorganisms. Unfortunately, as this was raw consumption data, we did not collect information on whether the administration was empiric or evidence based. This is important from an antimicrobial stewardship perspective, as it would determine the point at which they are mainly prescribed and would therefore facilitate decision making of ASPs. In addition, given that data of a single center is provided, this information may not be applicable to other settings with different features.

## 4. Materials and Methods

### 4.1. Study Design and Hospital Setting

This was a retrospective quasi-experimental before–after study conducted in a tertiary university hospital located in Barcelona, Spain. The hospital normally contains 450 beds, an 18-bed medical ICU, 7 beds in a surgical ICU, and 21 semi critical beds. This hospital also includes an active program for renal transplantation, and oncology and hematology wards.

### 4.2. Study Period and Study Population

To assess the potential impact of the COVID-19 pandemic of antimicrobial consumption in the hospital, we included two periods: pre-pandemic, which included the monthly consumption from January 2018 to February 2020; and the COVID-19 pandemic, with consumption data from March 2020 to June 2020. Although we acknowledge that the pandemic began on the 13th of March in our hospital, data for the whole month was included, as the informatic program does not allow one to split up the data into weeks. During this latter period, almost the entire hospital was dedicated to the care of patients with COVID-19.

### 4.3. Impact of the COVID-19 Pandemic

An ASP was implemented in our hospital from 1 January to 31 March 2011, composed of a multidisciplinary team that included specialists in infectious diseases, pharmacy, microbiology, and intensive care medicine [29]. This program included the following characteristics: we developed a computer application for the specific prescription of antimicrobials, which was added to the patient’s computerized medical record; and we selected 12 antimicrobials based on their ecologic and economic impact that underwent special control measures [29]. Among these measures, providers must justify their indications through the application form of the program; the information on the duration of treatment was mandatory, with automatic discontinuation of the antimicrobial on the day set by the prescriber physician, and immediate information of the cost of prescription was displayed. A member of the working group reviewed and reassessed these indications during the first 24 to 72 h and thereafter daily. The selected antimicrobials were linezolid, aztreonam, echinocandins (anidulafungin, caspofungin, micafungin), carbapenems (ertapenem, imipenem, meropenem), daptomycin, tigecycline, voriconazole, and liposomal amphotericin B [29].

During the first wave of the pandemic, the hospital increased its capacity to a total of 861 beds, 85 of which were for critically ill patients (expanded ICU). As has been described in other Spanish hospitals [6], during the COVID-19 pandemic, the ASP team modified its routine daily practice to attend pandemic management, so no ASP was formally available during this period. Local guidelines concerning the treatment of SARS-CoV-2 pneumonia, including the specific recommendations of the treatment with antibiotics, were developed by members of the infectious diseases, pharmacy, and ICU departments. Antibiotics were only recommended when a suspicion of bacterial co-infection was present (purulent sputum, inadequate clinical evolution) or in severe patients, mainly ceftriaxone 1–2 g once daily for 7 days. During the initial phases of the pandemic, the use of azithromycin 500 mg once daily, administered through oral or intravenous routes for three days, was recommended in combination with hydroxychloroquine, but this was discarded on 2 April. From an antibiotic prescription perspective, doctors from different specialties (including but not limited to dermatologists, cardiologists, endocrinologists, pulmonologists, oncologists, surgeons) were also in charge of patients admitted with SARS-CoV-2 pneumonia in the different hospital wards.

### 4.4. Antimicrobial Consumption

Consumption data was calculated using DDD/100 bed-days for both the overall hospital and ICU. This information was calculated independently by the Pharmacy Service based on dispensing rather than administration data, using our own computer system that estimated the DDD and days of therapy (DOT). For the calculation of the DDD/100 bed-days, the recommendations of the “Program for the Surveillance of Nosocomial Infection in Catalonia” (VINCat) [30,31] were used. Unlike in these recommendations, we calculated consumption data per month.

### 4.5. Statistical Analysis

The evolution of DDDs was analyzed through multiple linear regression with time as covariate and adding a change point on February to check possible trend changes after the beginning of pandemic period:

(1) $$DDD_{d}=\beta_{0}+\beta_{t}*t+\beta_{change}*ID_{change}$$

where *DDD_d_* is the accumulated *DDD* for each drug (*d*); t is the time rescaled from the beginning of the series (i.e., *t* = 1 for January 2018); and *ID_change_* is a binary variable defined as *ID_change_* = 1 if the measure is taken on February 2020 or later and 0 otherwise. *β*_0_, *β_t_*, and *β_change_* are the constants of the model and the coefficients for *t* and *ID_change_*, respectively.

Regression coefficients and *p*-values were reported. *p*-values were corrected for multiple comparisons using the Benjamini–Hochberg procedure and are described in the Supplementary Material.

STATA version 15.1 (StataCorp, College Station, TX, USA) was used for statistical analysis. *p*-values of 0.05 were considered statistically significant.

## 5. Conclusions

To conclude, we detected a worrisome global increase in the use of antimicrobials during the first wave of the COVID-19 pandemic, which was more evident in the ICU. This rise in the antimicrobial consumption showed a temporary pattern. Molecules that were used when there was a clinical suspicion of bacterial co-infection such as ceftriaxone or azithromycin presented their consumption peak in March, whereas broad-spectrum antibiotics achieved it in April–May. These findings underscore the importance of the availability of updated protocols for the approach to these pathologies and reinforce, more than ever, the need for ASPs to optimize antimicrobial use in hospitals. This is more necessary than ever, given the likely appearance of new waves and the threat of antimicrobial resistance.

## Data Availability

The data presented in this study are available on request from the corresponding author.

## Supplementary Materials

The following are available online at https://www.mdpi.com/2079-6382/10/2/132/s1, Table S1.

## References

[1] Programas de Optimización de Uso de los Antibióticos (PROA) | PRAN. https://www.resistenciaantibioticos.es/es/programas-de-optimizacion-de-uso-de-los-antibioticos-proa.

[2] CDC (2019). Core Elements of Hospital Antibiotic Stewardship Programs.

[3] Dellit T.H., Owens R.C., McGowan J.E., Gerding D.N., Weinstein R.A., Burke J.P., Huskins W.C., Paterson D.L., Fishman N.O., Carpenter C.F. (2007). Infectious Diseases Society of America and the Society for Healthcare Epidemiology of America guidelines for developing an institutional program to enhance antimicrobial stewardship. Clin. Infect. Dis..

[4] Dyar O.J., Beović B., Pulcini C., Tacconelli E., Hulscher M., Cookson B., Ashiru-Oredope D., Barcs I., Blix H.S., Buyle F. (2019). ESCMID generic competencies in antimicrobial prescribing and stewardship: Towards a European consensus. Clin. Microbiol. Infect..

[5] Rodríguez-Baño J., Paño-Pardo J.R., Alvarez-Rocha L., Asensio Á., Calbo E., Cercenado E., Cisneros J.M., Cobo J., Delgado O., Garnacho-Montero J. (2012). Programas de optimización de uso de antimicrobianos (PROA) en hospitales españoles: Documento de consenso GEIH-SEIMC, SEFH y SEMPSPH. Enferm. Infecc. Microbiol. Clin..

[6] Guisado-Gil A.B., Infante-Domínguez C., Peñalva G., Praena J., Roca C., Navarro-Amuedo M.D., Aguilar-Guisado M., Espinosa-Aguilera N., Poyato-Borrego M., Romero-Rodríguez N. (2020). Impact of the COVID-19 pandemic on antimicrobial consumption and hospital-acquired candidemia and multidrug-resistant bloodstream infections. Antibiotics.

[7] Youngs J., Wyncoll D., Hopkins P., Arnold A., Ball J., Bicanic T. (2020). Improving antibiotic stewardship in COVID-19: Bacterial co-infection is less common than with influenza. J. Infect..

[8] Uyeki T.M., Bernstein H.H., Bradley J.S., Englund J.A., File T.M., Fry A.M., Gravenstein S., Hayden F.G., Harper S.A., Hirshon J.M. (2019). Clinical Practice Guidelines by the Infectious Diseases Society of America: 2018 Update on Diagnosis, Treatment, Chemoprophylaxis, and Institutional Outbreak Management of Seasonal Influenza. Clin. Infect. Dis..

[9] Lansbury L., Lim B., Baskaran V., Lim W.S. (2020). Co-infections in people with COVID-19: A systematic review and meta-analysis. J. Infect..

[10] Adarsh Bhimraj A., Morgan R.L., Hirsch Shumaker A., Lavergne V., Baden L., Chi-Chung Cheng V., Edwards K.M., Gandhi R., Muller W.J., O’Horo J.C. (2020). Infectious Diseases Society of America Guidelines on the Treatment and Management of patients with COVID-19. Idsa.

[11] Hsu J. (2020). How covid-19 is accelerating the threat of antimicrobial resistance. BMJ.

[12] Clinical Management of COVID-19. https://www.who.int/publications/i/item/clinical-management-of-severe-acute-respiratory-infection-when-novel-coronavirus-(ncov)-infection-is-suspected.

[13] COVID-19 Treatment Guidelines Panel Coronavirus Disease 2019 (COVID-19) Treatment Guidelines. National Institutes of Health. https://www.covid19treatmentguidelines.nih.gov/.

[14] Ginsburg A.S., Klugman K.P. (2020). COVID-19 pneumonia and the appropriate use of antibiotics. Lancet Glob. Health.

[15] Zhu N.J., McLeod M., McNulty C.A.M., Lecky D.M., Holmes A.H., Ahmad R. (2021). Trends in Antibiotic Prescribing in Out-of-Hours Primary Care in England from January 2016 to June 2020 to Understand Behaviours during the First Wave of COVID-19. Antibiotics.

[16] Abelenda-Alonso G., Padullés A., Rombauts A., Gudiol C., Pujol M., Alvarez-Pouso C., Jodar R., Carratalà J. (2020). Antibiotic prescription during the COVID-19 pandemic: A biphasic pattern. Infect. Control Hosp. Epidemiol..

[17] Gonzalez-Zorn B. (2020). Antibiotic use in the COVID-19 crisis in Spain. Clin. Microbiol. Infect..

[18] Bassetti M., Kollef M.H., Timsit J.F. (2020). Bacterial and fungal superinfections in critically ill patients with COVID-19. Intensive Care Med..

[19] Langford B.J., So M., Raybardhan S., Leung V., Westwood D., MacFadden D.R., Soucy J.P.R., Daneman N. (2020). Bacterial co-infection and secondary infection in patients with COVID-19: A living rapid review and meta-analysis. Clin. Microbiol. Infect..

[20] Contou D., Claudinon A., Pajot O., Micaëlo M., Longuet Flandre P., Dubert M., Cally R., Logre E., Fraissé M., Mentec H. (2020). Bacterial and viral co-infections in patients with severe SARS-CoV-2 pneumonia admitted to a French ICU. Ann. Intensive Care.

[21] Garcia-Vidal C., Sanjuan G., Moreno-García E., Puerta-Alcalde P., Garcia-Pouton N., Chumbita M., Fernandez-Pittol M., Pitart C., Inciarte A., Bodro M. (2020). Incidence of co-infections and superinfections in hospitalized patients with COVID-19: A retrospective cohort study. Clin. Microbiol. Infect..

[22] Mayoral T.N., Gómez M.A.M., Rosselló G.A.M., Fuertes L.P., Benito E.C., García A.M.M., Martín A.B.M., Domingo A.O. (2020). Infección bacteriana/fúngica en pacientes con COVID-19 ingresados en un hospital de tercer nivel de Castilla y León, España. Enferm. Infecc. Microbiol. Clin..

[23] Langford B.J., So M., Raybardhan S., Mph B., Leung V., Soucy J.-P.R., Westwood D., Daneman N., Macfadden D.R., Langford Pharmd B.J. (2020). Antibiotic prescribing in patients with COVID-19: Rapid review and meta-analysis. Clin. Microbiol. Infect..

[24] Beovic B., Dousak M., Ferreira-Coimbra J., Nadrah K., Rubulotta F., Belliato M., Berger-Estilita J., Ayoade F., Rello J., Erdem H. (2020). Antibiotic use in patients with COVID-19: A “snapshot” Infectious Diseases International Research Initiative (ID-IRI) survey. J. Antimicrob. Chemother..

[25] Koehler P., Meis J.F., Ostrosky-zeichner L., Böll B., Cornely O.A., Eichenauer D.A. (2020). Covid-19/Influenza-Associated Pulmonary Aspergillosis—Management.

[26] Khor W.P., Olaoye O., D’arcy N., Krockow E.M., Elshenawy R.A., Rutter V., Ashiru-Oredope D. (2020). The need for ongoing antimicrobial stewardship during the COVID-19 pandemic and actionable recommendations. Antibiotics.

[27] Pulia M.S., Wolf I., Schwei R.J., Chen D., Lepak A.J., Schulz L.T., Safdar N. (2020). Antibiotic prescribing patterns for COVD-19 in two emergency departments with rapid procalcitonin. Infect. Control Hosp. Epidemiol..

[28] Mason C.Y., Kanitkar T., Richardson C.J., Lanzman M., Stone Z., Mahungu T., Mack D., Wey E.Q., Lamb L., Balakrishnan I. (2020). Exclusion of bacterial co-infection in COVID-19 using baseline inflammatory markers and their response to antibiotics Claire. J. Antimicrob. Chemother..

[29] Álvarez-Lerma F., Grau S., Echeverría-Esnal D., Martínez-Alonso M., Gracia-Arnillas M.P., Horcajada J.P., Masclans J.R. (2018). A Before-and-After Study of the Effectiveness of an Antimicrobial Stewardship Program in Critical Care. Antimicrob. Agents Chemother..

[30] Hernandez S., Horcajada J.P., Grau S., Echeverría D., Padullés A., Gimenez M., Oliva G., Boix L., Ferrer R., Limón E. (2019). INFORME DADES 2018 Monitoratge estandarditzat del consum hospitalari d’antimicrobians Programa VINCat Vigilància de les Infeccions Nosocomials als Hospitals de Catalunya.

[31] VINCat Program Programa de Vigilància de les Infeccions Nosocomials a Catalunya. Generalitat de Catalunya. Departament de Salut. http://catsalut.gencat.cat/ca/proveidors-professionals/vincat/.

