# Supplementary material for: Evolution of Antimicrobial Consumption During the First Wave of COVID-19 Pandemic
Source: Antibiotics (Basel). 2021 Jan 29;10(2):132. doi: 10.3390/antibiotics10020132 (PMC7911440; doi:10.3390/antibiotics10020132)
Supplement: Supplementary file 1 [file antibiotics-10-00132-s001.pdf]

Table S1. Evolution of drug consumption corrected for multiple comparisons in the analyses.

	**Hospital**				**Intensive Care Unit (ICU)**			
**Drug**	β_t	**P-value***	β_{change}	**P-value***	β_t	P-value*	β_{change}	P-value*
Ceftriaxone	−0.5	0.705	54.32	0.073	−0.23	0.617	20.49	0.053
Ceftaroline	0.26	0.414	11.77	0.073	0.15	0.102	−1.55	0.461
Carbapenems	1.49	0.368	−12.7	0.705	−0.31	0.461	26.10	0.014
Novel cephalosporins/β-lactamase inhibitors	0.71	0.073	−4.06	0.675	0.18	0.024	−1.70	0.354
Echinocandins	1.24	0.113	−20.6	0.321	0.19	0.270	−1.52	0.725
Vancomycin	−2.8	0.061	−7.44	0.705	−0.24	0.000	1.82	0.162
Daptomycin	2.65	0.073	11.15	0.705	0.06	0.827	18.09	0.014
Azithromycin	−1.2	0.662	63.13	0.267	−0.15	0.744	19.42	0.094
Linezolid	0.19	0.705	13.73	0.421	−0.27	0.162	9.18	0.050
Liposomal amphotericin B	0.18	0.457	−2.69	0.675	0.08	0.382	−0.31	0.852
Triazoles	0.32	0.705	17.6	0.563	−0.34	0.162	7.94	0.162
